# M. Dupuytren's Treatment of Specks on the Cornea

**Published:** 1830-04-01

**Authors:** 


M. Dupuytren's Treatment of Specks on the Cornea. From Dr. M'Lellan's translation of Ratier's work.

"For some years patients have flocked to the Hôtel-Dieu, afflicted with specks of the cornea, as they formerly resorted to Dessault, for the cure of chronic and scrophulous ophthalmia. The treatment pursued by M. Dupuytren is the following:—

"Detraction of blood from the arm, if there be much irritation.

"Leeches to the temple, if the irritation be less considerable.

"One or two mild purgatives at an interval of two or three days.

"After this a seton made of threads of cotton, and of a cylindrical form, introduced at the upper part of the neck, and passing several inches under the skin.

"Lastly, the insufflation of the subjoined powder on the eye, repeated morning and evening, by means of a quill, while the eyelids are kept separated.

"Oxidi Zinci Imp. Præp.

Sacchari Candi Albi

Submuriatis Hydrargyri } $\overline{aa}$ partes æquales.

"The eyes should neither be washed nor rubbed after the insufflation.

"When there does not exist any disease of the eyelids, nor inflammation of the conjunctiva, the insufflation of the powder generally suffices to resolve the specks.

"Those which are recent and slight, are completely dissipated in a few weeks by the insufflations. The specks that have existed longer, that are thicker and broader, usually give way in a month or six weeks, and specks that have occupied almost the whole cornea, covering the pupil, and intercepting entirely the passage of light into the eye, have been frequently seen to disappear completely in the course of a few months."

---

